# Philosophy and Medicine

**Published:** 1904-11-05

**Authors:** 


### Philosophy and Medicine.

In one of Lord Lytton's comedies, if we mistake not in "Not so Bad as We Seem," the play which he wrote to be performed by a distinguished company of amateurs for the benefit of the Guild of Literature and Art, an intrusive character is temporarily disposed of by being provided with the publications of Berkeley and of Locke. He is assured that the former will convince him that he has no material existence, and that the latter will satisfy him that he has no understanding. The medical profession was discoursed to on Friday by two of its most eminent members, Lord Kelvin and Sir James Crichton Browne, the former at St. George's Hospital and the latter in the rooms of the Medical Society of Edinburgh, and was assured by Lord Kelvin of the absolute distinctness of living from non-living matter, and by Sir James of the absolute distinctness of matter from mind. Lord Kelvin told his hearers that it was within the power of science to produce any crystalline compound, but that science had never succeeded in converting any combination of viscous fluid and electricity into a living cell, and that most certainly it never would succeed. From this apparently somewhat barren premise, however, he drew the eminently practical conclusion that, although the instruments of medicine and surgery, and the methods of instruction with regard to them, relate exclusively to non-living matter, their applications are entirely to living beings, and must throughout be guided by a knowledge of human nature, even although the study be one which finds no place in the official curriculum, and secures no recognition from examiners. Lord Kelvin described as "spiritual consolation" the effect so often produced by the visit and kindly words of the doctor in strengthening and encouraging the patient, although he disclaimed, of course, any desire to trespass upon the domain of theology. He told the advanced students, to whom (the occasion being a distribution of prizes) his observations were mainly addressed, that they could not go wrong if they allowed their natural feelings to prompt them at the sick bed, and that in this way they would be spiritual helps to their patients as well as physicians and surgeons. As men could not live on bread alone, patients could not be cured by drugs and splints alone; and he was glad to think that these were the views they held before them, and that they were preparing to go forth into the world as benefactors of the human race.

The dualism of Sir James Crichton Browne was not less positive, or, we might almost say, not less aggressive, than the biogenesis of Lord Kelvin; but it did not lend itself with the same readiness to the enforcement of any practical conclusion. When philosophers have agreed upon a definition of matter, and have become completely acquainted with its characters and properties, it will perhaps be possible to separate its province from that of "mind," and to establish some criterion of the share taken by one or other in the sustentation of the activities which collectively constitute life. Until this is done, the words must remain words only, and will be put up and knocked down by controversialists like ninepins on a village green; a fruitless pastime in which it may perhaps be regretted that Sir James should share. He was on much safer ground in the early part of his discourse, in which he enlarged upon the uses of medical societies in training their members to the practice of easy and fluent speech, the accomplishment in which he is himself so great a proficient; and in tracing back their power of rendering this service to the physiological principles underlying the successive development of different centres of motor activity. There is, as Sir James pointed out, "a tide in the affairs" not only of men, but of each motor centre, a time when it is most ready for activity and responsive to impressions. It follows from this principle that there is an "accepted time" for the cultivation of every variety of physical and intellectual acquirement, and that the work of education, in order to produce the best results, must be governed by continual recognition of the fact. If the highest degree of manual dexterity is ever to be acquired, the training of the hands must be commenced in early life, a fact which is perfectly well known to billiard players, although it is constantly ignored by school managers and other authorities, who allow the hands of children destined to lead industrial lives to dangle uselessly by their sides in class rooms. A little later comes the period of natural activity of the speech centre. A language acquired in childhood can for this reason always be spoken as if "to the manner born," while one only acquired during manhood, or even during adolescence, always betrays the speaker, even if it be only as the barbarian was detected at Athens, by the exquisite purity and correctness of his Greek. In like manner the power of what may be called public speaking can only be acquired in any high degree of excellence by practice during the period of early manhood, the period at which the formation of an intimate working partnership between the stores of the memory and the machinery for conveying them to an audience is most easily accomplished. For this reason Sir James urged upon his hearers that they should utilise to the full the opportunities afforded by their Society, and should accustom themselves to speak at its meetings. As it was said of Thiers in 1830, "Il parlait le premier, il parlait le dernier, il parlait toujours," so it should be said of everyone who really aspires to facility and distinction as a speaker.

